# Molecular and Cellular Mechanisms of Metformin in Cervical Cancer

**DOI:** 10.3390/cancers13112545

**Published:** 2021-05-22

**Authors:** Ya-Hui Chen, Po-Hui Wang, Pei-Ni Chen, Shun-Fa Yang, Yi-Hsuan Hsiao

**Affiliations:** 1 Women’s Health Research Laboratory, Changhua Christian Hospital, Changhua 500, Taiwan; 106317@cch.org.tw; 2 Department of Obstetrics and Gynecology, Chung Shan Medical University Hospital, Taichung 402, Taiwan; wang082160@gmail.com; 3 Institute of Medicine, Chung Shan Medical University, Taichung 402, Taiwan; peini@csmu.edu.tw; 4 School of Medicine, Chung Shan Medical University, Taichung 402, Taiwan; 5 Department of Medical Research, Chung Shan Medical University Hospital, Taichung 402, Taiwan; 6 Department of Obstetrics and Gynecology, Changhua Christian Hospital, Changhua 500, Taiwan

**Keywords:** cervical cancer, metformin, diabetes

## Abstract

**Simple Summary:**

The potential effects of metformin in terms of cancer prevention and therapy have been widely studied, and a number of studies have indicated its potential role in cancer treatment. Metformin exerts anticancer effects, alone or in combination with other agents, on cervical cancer in vitro and in vivo. Metformin might thus serve as an adjunct therapeutic agent for cervical cancer.

**Abstract:**

Cervical cancer is one of the major gynecologic malignancies worldwide. Treatment options include chemotherapy, surgical resection, radiotherapy, or a combination of these treatments; however, relapse and recurrence may occur, and the outcome may not be favorable. Metformin is an established, safe, well-tolerated drug used in the treatment of type 2 diabetes; it can be safely combined with other antidiabetic agents. Diabetes, possibly associated with an increased site-specific cancer risk, may relate to the progression or initiation of specific types of cancer. The potential effects of metformin in terms of cancer prevention and therapy have been widely studied, and a number of studies have indicated its potential role in cancer treatment. The most frequently proposed mechanism underlying the diabetes–cancer association is insulin resistance, which leads to secondary hyperinsulinemia; furthermore, insulin may exert mitogenic effects through the insulin-like growth factor 1 (IGF-1) receptor, and hyperglycemia may worsen carcinogenesis through the induction of oxidative stress. Evidence has suggested clinical benefits of metformin in the treatment of gynecologic cancers. Combining current anticancer drugs with metformin may increase their efficacy and diminish adverse drug reactions. Accumulating evidence is indicating that metformin exerts anticancer effects alone or in combination with other agents in cervical cancer in vitro and in vivo. Metformin might thus serve as an adjunct therapeutic agent for cervical cancer. Here, we reviewed the potential anticancer effects of metformin against cervical cancer and discussed possible underlying mechanisms.

## 1. Introduction

Cervical cancer, one of the major gynecologic malignancies, is a global public health issue [1]. Its prognosis and optimal management are primarily determined based on the stage of the disease [2]. Treatment options include chemotherapy, surgical resection, radiotherapy, or a combination of these treatments; however, relapse and recurrence may occur, and the outcome may not be favorable [2]. Gaining a better understanding of related molecular biological factors could assist in the development of better molecular treatment options.

Metformin, derived from galegine, is a natural product from the plant *Galega officinalis* [3], and has been widely-used to treat diabetes since the 1950s [4]. Although its use was discontinued for some time in light of it causing lactic acidosis, metformin was found to be safe and effective in lowering glucose levels, and thus its use was reinstated in 1995 [4]. Metformin is a low-cost drug indicated for type 2 diabetes, and does not induce hypoglycemia or weight gain [4]. Sixty years of clinical experience have yielded almost no safety concerns for metformin [4]. A systematic review including 17 observational studies concluded that metformin use reduces all-cause mortality in populations with type 2 diabetes and congestive heart failure, moderate to severe chronic kidney disease, or chronic liver disease with hepatic impairment [5]. Moreover, metformin can be safely combined with other antidiabetic agents [6].

Recently, researchers investigated the potential anticancer role of metformin in non-diabetic patients with cancer, such as lung cancer, breast cancer and prostate cancer; however, the results are controversial [7]. The role that metformin may play in non-diabetic women with cervical cancer remains unclear since metformin is typically given to only diabetic patients and the typical cervical cancer population is young and nondiabetic [8]. In Takiuchi et al.’s study [8], the authors examined the association of metformin use and survival outcome in women with cervical cancer by stratifying patients into diabetic metformin users, 68; diabetic metformin nonusers, 42; nondiabetic patients, 673. There were two metformin users, among the 673 nondiabetics, excluded in that study for not being diabetic. More studies are needed to clarify the role or metformin in non-diabetic women with cervical cancer.

The potential role that metformin may play in prostate cancer has been investigated [9,10,11]. Metformin therapy may decrease the incidence of prostate cancer but there is no association between treatment and mortality or recurrence [9]. However, a systematic review and meta-analysis study revealed the association of metformin with lower prostate cancer recurrence in type 2 diabetes [10]. Metformin has been reported to reduce the level of androgen receptor (AR) protein in AR-positive cell lines and to suppress the AR signaling pathway via down-regulation of AR mRNA. This observation supports the role of metformin as a potential adjunctive therapy to androgen deprivation therapy (ADT) [12]. Ongoing clinical trials evaluating metformin as an adjuvant therapy are necessary [11].

Recent studies have showed the abilities of metformin to activate the AMPK pathway, increase cellular apoptosis, and inhibit the mTOR/AKT pathway [13,14]. The potential effects of metformin in terms of cancer prevention and therapy have been widely studied [15]. Diabetes, possibly associated with an increased site-specific cancer risk [6,16], may contribute to the initiation and progression of specific types of cancer. Furthermore, diabetes linked to poorer survival of patients with cervical cancer was revealed by the meta-analysis [17]. Hanprasertpong et al. showed that metformin use in cervical cancer patients with type 2 diabetes mellitus was associated with improved disease-free survival [18]. However, not all the studies are in progress are on the prognostic significance of metformin in cervical cancer patients. Takiuchi et al. demonstrated no association between metformin use and the survival outcome of women with cervical cancer [8]. An epidemiologic study evaluated the association between metformin use and oncological outcomes in patients with cervical cancer and type 2 diabetes, and revealed that patients receiving metformin had a lower recurrence rate than those not receiving metformin [18]; moreover, non-use of metformin (hazard ratio, 1.89; *p* = 0.037) was an independent adverse prognostic factor of 5-year disease-free survival, but not of overall survival [18]. Several epidemiological studies have revealed an association between type 2 diabetes and an increased risk of many types of cancer, including cervical cancer [19]; furthermore, type 2 diabetes might have an impact on prognosis in patients with cervical cancer [19].

The association of metformin with cancer risk has been investigated. Metformin may reduce the risk of cancer in patients with type 2 diabetes [20,21], and several studies have demonstrated an association between metformin use and prognosis or mortality in patients with cancer. Among older women with diabetes and cervical cancer, cumulative doses of metformin may be associated with a significant decrease in mortality [22]; however, a recent study indicated that the survival outcome in women with cervical cancer was not associated with metformin use [8], while another study showed that metformin may reduce the risk of gynecological cancers [23]. Metformin significantly reduced the risk of cancer (RR = 0.27, 95% CI = 0.17–0.41) and the overall risks of ovarian (RR = 0.18, 95% CI = 0.12–0.28) and cervical cancer (RR = 0.60, 95% CI = 0.43–0.83) in an Asian population [23].

## 2. Mechanism of Metformin in Cervical Cancer

The mechanism most frequently-proposed to underlie the diabetes–cancer association is insulin resistance, which leads to secondary hyperinsulinemia; furthermore, insulin may exert mitogenic effects through the insulin-like growth factor 1 (IGF-1) receptor [24,25], and hyperglycemia may worsen carcinogenesis through the induction of oxidative stress [26,27]. The anticancer effects of metformin remain under-investigated. Antitumor effects of metformin have been proposed [28]. At the extracellular level, metformin may reduce the circulating insulin level and activate the immune system; intracellularly, metformin may activate the liver kinase B1 (LKB1)/AMP-activated protein kinase (AMPK) pathway, inhibit protein synthesis, induce cell-cycle arrest and apoptosis, and reduce IGF-1 and insulin-mediated signaling [28]. AMPK activators are used in the treatment of type 2 diabetes and cancer [29]. Metformin has anticancer effects on both AMPK-independent and -dependent actions [30]. Metformin may improve the immune response to cancer cells [30]. Metformin has been demonstrated to possess anticancer activity, both in vitro and in vivo [31]. Metformin exhibits anticancer actions via both direct and indirect effects [32]: the indirect effects include reduction of circulating glucose and insulin levels, and suppression of tumor progression by indirectly modulating IGF-1 signaling, which promotes tumor cell growth [33], while the direct effects include initiation of cell-cycle arrest, suppression of epithelial-mesenchymal transition (EMT), and inhibition of tumorigenesis and cancer progression [33].

Metformin may inhibit cancer cells through various other mechanisms, such as mTOR pathway inhibition and autophagy induction [34]. Understanding the molecular mechanisms underlying the anticancer effect of metformin is crucial. Here, we reviewed the potential anticancer effects of metformin against cervical cancer and discussed possible underlying mechanisms.

**Figure 1:**
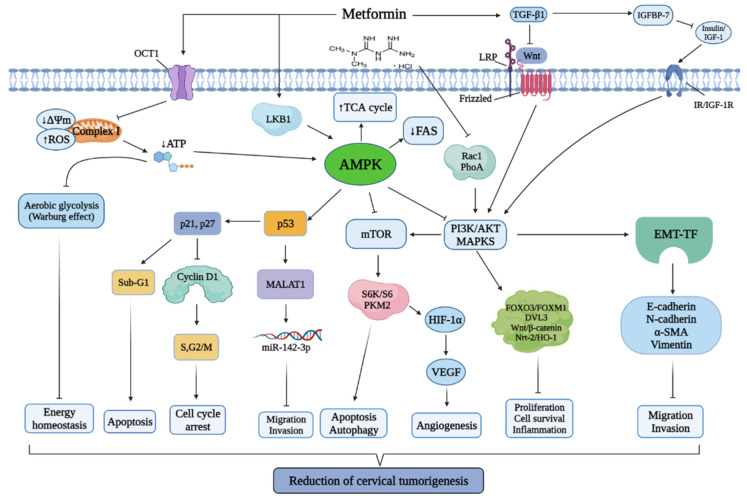
Anticancer molecular mechanisms of metformin in cervical cancer. Metformin mainly modulates AMPK activation through LKB1, which activates or inactivates various downstream signaling targets, such as p53, mTOR, PI3K/Akt, MAPKs, TGF-β1, IGF-1, and transcription factors (FOXO3a, DVL3, Wnt/β-catenin, HIF-1α). The activation of these signaling pathways induce cell cycle arrest, apoptosis, and autophagy that inhibit tumorigenesis in the cervical cancer cell while suppressing cellular migration, invasion, angiogenesis, inflammation, and cell proliferation.

Several studies have investigated the potential use of metformin for the treatment of cervical cancer. Metformin has been implied to induce tumor suppression and attenuate cancer growth [28]. Studies examining the effects of metformin on cervical cancer cells are listed in Table 1. Figure 1 summarizes the anticancer molecular mechanisms of metformin on cervical cancer cell. With regards to the action of metformin in diabetes, it was revealed that metformin activates AMPK, which leads to targeting of rapamycin complex 2 (TORC2) phosphorylation and results in the blocking of translocation and transcription of genes related to gluconeogenesis [35]. LKB1 exerts an effect that is biochemically sufficient to activate AMPK [35].

### Effects of Metformin on Cervical Cancer Cells

The direct anticancer effects of metformin are essentially mediated by AMPK-dependent (decreasing mTOR, c-MYC and NFκB, and increasing p53 phosphorylation) and AMPK-independent (decreasing ROS, increasing mTORC1, decreasing cyclin D1, increasing autophagy and apoptosis of cancer cells) mechanisms [32,33].

AMPK acts as a regulator of the response to the low energy expressed in cells for the balance of cellular ATP and AMP concentrations [35]. LKB1 serine/threonine kinase biochemically activates AMPK [35]. LKB1 may act as a regulator of metabolism, and LKB1/AMPK signaling plays a role in protection against apoptosis [35]. The actions of LKB1/AMPK appear to contribute to the anticancer property of metformin, specifically in patients with diabetes [35].

A23187, an alternative pharmacological AMPK activator, was introduced to inhibit cervical cancer cell growth via activation of Ca^2+^/calmodulin-dependent protein kinase kinase β (CaMKKβ), another upstream kinase of AMPK [48]. A23187 exerted an antiproliferative effect on cervical cancer cells through suppressing AMPK/mTOR signaling activity. These data suggested that A23187 could be a potential therapeutic antiproliferation drug for use in LKB1-deficient cancer cells [48].

The adjuvant role of metformin has been investigated in combination treatments for several kinds of cancer, such as non-small-cell lung cancer (NSCLC) [49] and breast cancer [40] through varied mechanisms. The enhancement of chemotherapeutic cytotoxicity towards NSCLC by metformin was investigated [49]. Cisplatin-based chemotherapy remains the first-line drug for NSCLC patients without epidermal growth factor receptor (EGFR) mutations; however, drug resistance can develop during therapy through varied mechanisms, such as mediation by activation of the IL-6/signal transducer. A study was performed to examine the correlation between STAT3 phosphorylation and cisplatin cytotoxicity in lung adenocarcinoma cells [49]. Metformin suppressed STAT3 activation through ROS-related and autocrine IL-6 production-related pathways [49].

The effect of cisplatin combined with metformin on triple-negative breast cancer (TNBC) was investigated [40]. Cisplatin in combination with metformin exhibited a greater ability to decrease cell viability and the metastatic effect as compared with cisplatin alone [40]. A synergistic anticancer effect was found in a murine model of 4T1 breast cancer in vivo. Metformin was observed to overcome resistance to cisplatin by decreasing the RAD51 protein stability and increasing its ubiquitination [40].

Metformin was found to amplify chemotherapy-induced AMPK activation in cancer cell lines and severe combined immunodeficient (SCID) xenografted mice [50]. Activation of AMPK and reduction of mTOR signaling is one mechanism involved in the action of metformin [50]. Chemotherapy agents that exert genotoxic stress and induce p53 activity can cross-talk with the AMPK/mTOR pathway, and metformin amplifies chemotherapy-induced AMPK activation [50].

A combination of metformin and paclitaxel enhanced the inhibition of antitumor growth by increasing the number of cell cycle arrests and increasing apoptosis in tumor-bearing mice [50]. Metformin inhibition of cell viability and migration, and induction of cell-cycle arrest and apoptosis in cervical cancer cell lines (CaSki and HeLa) were reported [36]. This study showed that metformin reduced cervical cancer cell viability and migration, induced apoptosis and cell-cycle arrest, activated the AMPK/p53 signaling pathway and decreased PI3K/AKT signaling [36]. AMPK signaling pathway activity was found to be related to metformin-induced cytotoxicity and apoptosis in cervical cancer cells; thus, these effects on apoptosis and cell-cycle arrest were induced through the AMPK/p53 and I3K/AKT/mTOR signaling pathways [36].

Metformin targets the PI3K/Akt and p53 pathways, and modulates the antitumor immune response in cervical cancer, as reported by Xia et al. [37]. In a study examining the effects of metformin in HeLa cervical cancer and ZR-75-1 breast cancer cell lines, metformin induced HeLa cell death through downregulation of p53 protein [51]. In a study performed by Xiao et al., cervical cancer cells, with intact LKB1, were sensitive to metformin, and presented an integral AMPK-mTOR signaling response [38]. Metformin reduced the cancer cell viability and inhibited cancer cell growth via the activation of LKB1-AMPK signaling [38]. Metformin can be used to treat gynecological tumors, as it causes activation of AMPK, enhancement of phosphorylation of LKB1, and inhibition of mammalian target of rapamycin [52].

The effect of metformin on migration of HeLa cervical cancer cells was examined [39]. Metformin treatment inhibited migration of cervical cancer cells, and the formation of filopodia and lamellipodia was depleted. The mechanism of a suppressive effect on cervical cancer cell migration was mediated by inhibition of filopodia and lamellipodia formation via suppressing the regulatory proteins FAK, Akt, and downstream Rac1 and RhoA [39].

The underlying mechanism of metformin regarding induction of cell-cycle arrest in cancer cells was examined in a previous study, and the researchers demonstrated that metformin inhibits cervical cancer cell proliferation through decreased AMPK O-GlcNAcylation [53]. Increasing levels of O-GlcNAc transferase (OGT) and O-linked N-acetylglucosamine (O-GlcNAc) were found in cervical cancer cells, but the effects were reversed by metformin treatment [53]. In comparison with untreated cells, O-GlcNAcylated AMPK was decreased and the level of phospho-AMPK was increased in cells treated with metformin [53].

The effect of metformin in terms of epigenetic dysregulation in cervical cancer was investigated previously [41]. Long non-coding RNA MALAT1, elevated in various types of cancer, promotes tumor growth and metastasis [54,55]. It was reported that microRNA (miR)-142-3p inhibits cervical cancer cell by targeting FZD7 [56]. A low level of miR-142-3p was associated with an advanced disease stage, lymph node metastasis and the depth of cervical invasion; patients with a low miR-142-3 expression had poorer progression-free survival and overall survival [57]. In the study by Xia et al., metformin suppressed cervical cancer cell migration and invasion [41]. It was found in that study that miR-142-3p was significantly upregulated, but HMGA2, lncRNA and MATAL1 were suppressed by metformin [41]. Metformin disrupts the MALAT1/miR-142-3p sponge, resulting in decreased migration and invasion of cervical cancer cells [41].

FOXM1, a proliferation-associated transcription factor, activates proliferation by promoting S-phase entry and M-phase entry [58], and has been implicated in tumorigenesis and suggested to be related to tumor initiation and progression [58]. Metformin activates AMPK and also counteracts the function of FOXM1 to inhibit cervical cancer cell growth [42].

The role of DVL3 in the inhibition of cervical cancer cells by metformin was studied [43]. DVLs, important signal transduction molecules, mediate Wnt/β-catenin signaling activity to influence cell growth [43]. DVL3 enhances the proliferation of cervical cancer cells, and metformin reduces DVL3 [43]. Metformin inhibits the growth of cervical cancer cells by impairing DVL3 protein synthesis or partially promoting the proteasomal degradation of DVL3 [43].

Metformin has been found to be related to epithelial-to-mesenchymal transition (EMT) in cervical cancer cells [44]. EMT plays an important role in tumorigenesis; it contributes to cells with migratory and invasive properties, and promotes cancer progression through a variety of mechanisms [59]. EMT may be a determinant of chemosensitivity or chemoresistance [60]. One study examined cells cultured with transforming growth factor beta 1 (TGF-β1) to induce EMT and treated cells with or without metformin, and metformin was observed to inhibit EMT [44]. The results showed that metformin inhibited TGF-β1-induced apoptosis, migration and proliferation of cervical carcinoma cells [44]. These effects were through the mTOR/p70s6k/PKM2 signaling pathway, which is involved in regulating PKM2 expression [44].

Metformin has been reported to inhibit heme oxygenase-1 (HO-1) expression in cancer cells, including cervical cancer HeLa cells [45]. HO-1 acts as a sensor and regulator of oxidative stress, regulating angiogenesis and cell proliferation [61,62]; however, HO-1 may also play a role in tumorigenesis and promote angiogenesis in cancer development [63,64]. HO-1 overexpression can confer tumor cell resistance to apoptosis [65], and inhibition of HO-1 expression may increase the responsiveness of tumor cells such as prostate cancer [66] and pancreatic cancer cells [67] to radiotherapy and chemotherapy. In a study examining the effects of metformin on cancer cells, metformin was observed to strongly suppress HO-1 expression in cervical cancer HeLa cells, and HO-1 inhibition provided metformin with antiproliferative effects [45]. The suppression of HO-1 by metformin is independent of AMPK [45].

The role of LKB1 in the response of cervical cancer cells to metformin was studied [38]. LKB1 was initially identified as the causal mutation in Peutz-Jeghers Syndrome (PJS) [68], which is a rare cancer-susceptibility syndrome [69]. LKB1, a multi-functional protein, is expressed in many cell types and tissues; it plays important roles in cell metabolism, regulating cell growth, cell proliferation, cell polarity and energy balance [49,70,71]. LKB1 expression and the integrity of LKB1-AMPK signaling was examined in cervical cancer cells under metformin treatment [38], and the LKB1-intact cervical cancer cells exerted an integral AMPK-mTOR signaling response [38].

The differentially-expressed proteins induced by metformin were examined using iTRAQ-based quantitative proteomic analysis [46]. Metformin was found to mainly regulate the insulin signaling pathway, and upregulated the expression of tumor suppressor IGFBP7 to inhibit the invasion and proliferation of cervical cancer cells.

Chemotherapy is one of the standard therapies for cervical cancer, but it frequently gives rise to adverse effects and drug resistance, or may not be an effective method of treatment. Several studies have examined the combination of therapeutic agents with doxorubicin in terms of enhancing the antitumor effects of doxorubicin [72,73,74].

To compare the antitumor effects of metformin and doxorubicin, Yudhani et al. [47] conducted a study using HeLa cells treated with various doses of metformin and doxorubicin as a positive control [47]. Metformin inhibited cell proliferation and induced apoptosis by modulating the expressions of cyclin D1 and p53 [47]. HeLa cells were treated with various doses of metformin and doxorubicin; the results showed that metformin was able to inhibit the proliferation of HeLa cells, and HeLa cells treated with metformin had a lower cyclin D1 expression than cells without metformin treatment (*p* = 0.001) [47]. The cancer cells treated with a metformin dose of 30 mM or greater exhibited a significantly increased p53 expression (*p* < 0.001) [47]. Treatment of HeLa cells with all doses of metformin induced apoptosis to a significant degree [47]. Metformin caused inhibition of cell proliferation and induction of apoptosis through modulation of the expressions of cyclin D1 and p53 [47].

Action of metformin involving mitochondria has been taken into consideration. Marini et al. reported that metformin directly inhibited the enzymatic function of hexokinase in breast cancer [75]. Andrzejewski et al. demonstrated that metformin decreased mitochondrial respiration and reduced glucose metabolism through the citric acid cycle [76]. Moreover, it has been shown that metformin suppressed the proliferation of breast cancer cells and colon cancer cells, which may be due to the modulation on cell energy metabolism [77,78].

## 3. Combination of Metformin with Therapeutic Agents Induced Anti-Cervical Cancer Effects

Metformin has been combined with therapeutic agents in order to induce anti-cervical cancer effects, as shown in Table 2.

### 3.1. Metformin Combined with Carboplatin

Carboplatin, an analogue of cisplatin, causes less nephrotoxicity, neurotoxicity, emesis and ototoxicity than cisplatin at doses conferring equivalent antitumor effects [85,86]. Carboplatin is integrated into combination regimens to treat cervical cancer; it is also used as part of combined-modality regimens prior to radiotherapy [85]. The effects of metformin in combination with carboplatin in terms of inhibiting cervical cancer HeLa cells were investigated [79]. HeLa cells were treated with metformin and/or carboplatin at different doses, and the results showed that metformin combined with carboplatin significantly reduced the HeLa cell viability and increased the number of nuclear fragments as compared with cells without drug treatment (*p* < 0.05) [79]. Metformin in combination with carboplatin enhanced the apoptotic rate and decreased the mitochondrial membrane potential as compared with the no-drug treatment group (*p* < 0.05) [79]. That study revealed that metformin enhanced the inhibitive effects of carboplatin on HeLa cell proliferation and increased the sensitivity of HeLa cells to treatment with carboplatin through activating the mitochondrial-associated apoptosis signaling pathway [79]. Surgery is the preferred therapy for patients with early-stage cervical cancer, but the reproductive function could be damaged; chemotherapy is the usual approach for advanced-stage or metastatic cervical cancer [87]. Cytotoxic chemotherapy has been shown to have impacts on the outcome of patients with recurrent, persistent or metastatic cervical cancer [88].

Platinum remains the first-line anticancer drug to kill tumor cells [89]. Carboplatin, a platinum analogue, has undergone widespread clinical testing in various cancers as a replacement for cisplatin [90]. In preclinical systems, carboplatin exhibited lesser toxicity and equivalent biochemical selectivity as compared with cisplatin [90]. In the clinical setting, the recurrence of tumors may be induced by chemotherapeutic drug resistance [91]. The disease usually relapses and becomes refractory in patients undergoing single-agent platinum therapy [92], and an appropriate combination of drugs targeting specific mechanisms may reduce cancer cell growth and improve survival. In a study of the effects of metformin in combination with carboplatin on HeLa cells, the inhibitive effects of carboplatin on cell proliferation were significantly enhanced and the apoptotic rate was increased [79].

### 3.2. Metformin Combined with Caffeic Acid

Caffeic acid (trans-3,4-dihydroxycinnamic acid) is a potent antioxidant abundant in herbs, coffee, red wine, berries and fruit. Caffeic acid reduced the viability and migration rate of oral carcinoma cells that were exposed to low concentrations of ethanol (between 2.5 and 10 mmol/L) [93]. Caffeic acid may exert regulatory effects in cells and possesses chemopreventive effects. Caffeic acid exhibited a protective effect on paclitaxel-treated lung cancer cells mediated via the NF-κB signaling pathway [94]. Caffeic acid and 5-caffeoylquinic acid demonstrated inhibitory effects on the growth of colon adenocarcinoma cells, which may act through modulating the cell cycle and increasing apoptosis in cancer cells [95].

Research has been performed to examine the effects of metformin and caffeic acid alone or in combination on aggressive metastatic cervical cancer cells (HTB-34 cells) [80]. Caffeic acid treatment results in increased oxidative stress and sensitization of cancer cells, acting on bioenergetics and cell biosynthesis and rendering HTB-34 cells more susceptible to metformin, which led to inhibition of neoplastic cells [80]. Disruption of the bioenergetics of cancer cells C-4I and HTB-35/SiHa by a combination of caffeic acid and metformin was investigated [81]. Researchers employed cervical cancer cells C-4I and HTB-35/SiHa in order to gain a greater understanding of the abilities of caffeic acid, metformin and the combination of both to disrupt the bioenergetics of cancer cells [81].

Caffeic acid activates AMPK and diminishes lipid de novo biosynthesis in C-4I cells [81], while metformin decreases HIF-1*a* protein stability under hypoxia and lowers the level of HIF-1α-induced glycolytic enzymes in HTB-35 cells [81]. Both caffeic acid and metformin exert disruptive effects on energy homeostasis, regulation of the oxidative metabolism and glycolysis in cervical cancer cells of a specific metabolic phenotype [81].

The effects of metformin and caffeic acid in terms of inhibiting cervical cancer cells C-4I and HTB-35/SiHa by acting on varied molecular targets were studied [82], and it was found that these two molecules may be of potential use in metastatic cervical cancer therapy [82].

### 3.3. Metformin Combined with Caffeic Acid and Cisplatin

Chemotherapy with cisplatin, a small-molecule platinum compound, appears to be effective in patients with advanced or recurrent cervical cancer [96]; however, cisplatin resistance may develop, which may be a result of genetic and multiple epigenetic changes [97]. A combination regimen is the standard chemotherapy to treat recurrent or metastatic cervical cancer [98]. Co-treatment with metformin, caffeic acid and cisplatin was administered to suppress cervical cancer cells [83]. Metformin and caffeic acid augmented the effects of cisplatin against quiescent tumor cells, which involved reprogramming of the cell cycle [83]. Metformin and caffeic acid co-treatment decreases SiHa cells growth and sensitizes cervical cancer cells to the action of cisplatin [83]. Co-treatment of SiHa cells with metformin, caffeic acid and cisplatin inhibits the cell cycle by decreasing the number of cells in the G0 phase [83]. These results suggested new cisplatin-based selective strategies for cervical cancer treatment [83].

### 3.4. Metformin Combined with Nelfinavir

The anti-tumor effects of metformin combined with nelfinavir on cervical cancer cell xenograft nude mice were investigated by Xia et al. [84]. In addition, the anti-cancer properties of nelfinavir, an HIV protease inhibitor, have been explored [99]. The effects of nelfinavir on NSCLC cells and tumor xenografts in nude mice were investigated [100]; moreover, it was found that the effect of docetaxel on inhibiting the growth of NCI-H460 and -H520 cells could be enhanced by nelfinavir [100]. Nelfinavir promotes apoptosis and inhibits growth of human melanoma cells in the G(1) phase [101], and an activity of nelfinavir against cervical cancer cells was observed [102]. Nelfinavir promoted apoptosis in a way that was attributed to the enhancement of mitochondrial reactive oxygen species (ROS) production [102].

Nelfinavir exerts anticancer effects, including cell-cycle arrest, cell death, endoplasmic reticulum stress, unfolded protein response, autophagy, inhibition of proteasomes, and downregulation of Akt signaling, alters the tumor microenvironment, and can be used in multidrug efflux pumps [99]. The effects of metformin in combination with nelfinavir include synergistic effects against growth, inhibition, migration and invasion, upregulating the expressions of ROS, p53 and p2, and downregulating the expression of PI3K(p110α) in cervical cancer cell xenograft nude mice [84]. In general, combining two active molecules with the same target in a signal pathway may result in antagonist effects, while combining two active molecules with different targets in a signal pathway may lead to synergistic effects [103]. Nelfinavir may target Akt and downregulate the P13K/Akt/mTOR signal pathway [104], while metformin targets mTOR and downregulates the P13K/Akt/mTOR signal pathway [105]. In one study, metformin in combination with nelfinavir exerted a synergistic action in terms of downregulating the P13K/Akt/mTOR signal pathway and suppressing the protein expression of P13K [84].

### 3.5. Clinical Trials of Metformin in Cervical Cancer

Information involving clinical trials using metformin in non-diabetic women with cervical cancer was obtained (http://clinicaltrials.gov/, accessed on 19 May 2021). A past clinical trial (NCT02394652), a multicenter phase II randomized trial, involved metformin with standard chemoradiation (cisplatin given with external beam radiation) and active comparator of standard chemoradiation for non-diabetic patients with locally advanced cervix cancer. The study aimed to determine if metformin decreases tumor hypoxia measured on positron emission test (PET) performed with a hypoxia dye FAZA. This study was completed in January 2021. Results might be achieved. An ongoing clinical trial (NCT04275713), involves experimental standard cisplatin-based chemoradiotherapy +/− metformin and active comparator standard chemoradiotherapy for non-diabetic patients with locally advanced cervix cancer. The study is to determine if there are metformin dependent changes in hypoxia-related gene expression. Promising outcomes might be anticipated to improve management in nondiabetic cancer patients.

## 4. Conclusions

Metformin is an established, safe, well-tolerated drug for the treatment of type 2 diabetes. A number of studies have indicated a potential role of metformin in cancer treatment [106]. Furthermore, combining current anticancer drugs with metformin may increase their efficacy and diminish adverse drug reactions [106]. Accumulating evidence indicates that metformin exerts anticancer effects [107], alone or in combination with other agents, on cervical cancer, in vitro and in vivo. Metformin might thus serve as an adjunct therapeutic agent for cervical cancer. Future studies should be performed to elucidate the mechanisms underlying the anticancer effects of metformin, and to assess its safety and efficacy in patients with cervical cancer.

## Figures and Tables

**Table 1:** Efficacy of metformin on cervical cancer cells.

Cell Lines/Animal Models	Anti-Cancer Effects	Mechanisms	Reference
CaSki, C33A, HeLa	↑ apoptosis ↓ migration	↑ AMPK/p53 ↓ PI3K/AKT	Chen et al. [36]
SiHa, HeLa BALB/c nude mice	↓ proliferation ↓ tumor growth in xenografts	↑ p53, MICA and NK cell cytotoxicity ↓ PI3K/AKT	Xia et al. [37]
C33A, Me180, CaSki, HeLa, HT-3, MS751	↑apoptosis ↑autophagy	↑ LKB1-AMPK-mTOR	Xiao et al. [38]
HeLa	↓ migration	↑ FAK/Akt ↓ Rac1 and PhoA protein expression	Hakimee et al. [39]
HeLa	↓ proliferation ↑apoptosis ↑ sub-G1 arrest	↓ AMPK O-GlcNAcylation ↑ p21 and p27 levels	Kim et al. [40]
SiHa, HeLa BALB/c nude mice	↓ invasion and migration ↓ tumor growth in xenografts	↓ MALAT1 expression ↑ miR-142-3p expression	Xia et al. [41]
HeLa, CaSki, C33A, SiHa	↓ proliferation	↑ AMPK ↓ AKT/FOXO3a/FOXM1	Yung et al. [42]
HeLa, SiHa, C33A, CaSki, C41	↓ proliferation	↓ DVL3 protein synthesis ↑ AMPK activation ↓ Wnt/β-catenin signaling	Kwan et al. [43]
HeLa, SiHa	↑ apoptosis ↓ proliferation ↓ migration	↓ TGF-β1-induced EMT effects ↓ mTOR/p70s6k/PKM2	Cheng et al. [44]
HeLa	↓ proliferation	↓ HO-1 protein expression ↓ Raf-ERK-Nrf2 ↑ AMPK-independent mechanisms	Do et al. [45]
HeLa, SiHa BALB/c nude mice	↓ proliferation ↓ invasion and migration ↓ tumor growth in xenografts	↑ IGFBP7 protein expression Regulates the insulin signaling pathway	Xiao et al. [46]
HeLa	↑ apoptosis ↓ proliferation ↑ S and G2/M arrests	↓ Cyclin D1 expression ↑ p53 expression	Yudhani et al. [47]

↓: inhibit or decrease; ↑: induce or increase.

**Table 2:** Metformin in combination with therapeutic agents exhibiting anti-cervical cancer effects.

Combined Therapeutic Agent	Cell Lines/Animal Models	Anti-Cancer Effects	Mechanisms	Reference
Carboplatin	HeLa	↑ apoptosis ↓ proliferation	↑ Nuclear fragments formation ↓ Mitochondrial membrane potential	Tang et al. [79]
Caffeic acid	HTB-34	↑ apoptosis ↓ proliferation ↑ S and G2/M arrests	↑ AMPK ↑ mitochondrial ROS ↑ TCA cycle ↑ Fatty acids *de novo* synthesis	Tyszka-Czochara et al. [80]
Caffeic acid	C-4I, HTB-35/SiHa	↑apoptosis ↓ proliferation ↓ energy homeostasis	↓ c-Myc, BAX and cyclin-D1 expression ↓ HIF-1α and Warburg effect ↑ mitochondrial ROS ↑ AMPK regulating oxidative metabolism/glycolysis	Tyszka-Czochara et al. [81]
Caffeic acid	C-4I, HTB-35/SiHa	↓ proliferation ↓ migration	↑ Epithelial adhesive markers ↓ Mesenchymal transcription factors regulating EMT	Tyszka-Czochara et al. [82]
Caffeic acid	HTB-35/SiHa	↑ apoptosis ↑ cisplatin anti-cancer action	↑ AMPK ↑ mitochondrial ROS ↑ TCA cycle ↓ novo unsaturated fatty acid synthesis	Tyszka-Czochara et al. [83]
Nelfinavir	HeLa, SiHa, CaSki BALB/c nude mice	↑ apoptosis ↓ proliferation ↓ invasion and migration ↓ tumor growth in xenografts	↑ ROS, p53 and p21 expression ↓ PI3K (p110α)	Xia et al. [84]

↓: inhibit or decrease; ↑: induce or increase.

## Data Availability

Not applicable.

## Abbreviations

AMPK	AMP-activated protein kinase
CaMKKβ	Ca2+ /calmodulin-dependent protein kinase kinase β
EMT	epithelial-mesenchymal transition
HIF-1α	hypoxia-inducible factor-1α
HO-1	heme oxygenase-1
IGF-1	insulin-like growth factor 1
IGFBP7	Insulin Like Growth Factor Binding Protein 7
LKB1	liver kinase B1
MALAT1	metastasis associated lung adenocarcinoma transcript 1
MAPK	mitogen-activated protein kinase
OCT-1	organic cation transporter-1
ROS	reactive oxygen species
TGF-β1	transforming growth factor beta 1
TORC2	rapamycin complex 2
VEGF	vascular endothelial growth factor
